# FYB1-targeted modulation of CAPG promotes AML progression

**DOI:** 10.1007/s11010-024-04992-4

**Published:** 2024-05-03

**Authors:** Wenyuan Liu, Hongli Yin, Zhiwei Xie, Fang Fang, Jinhua Chu, Linhai Yang, Lingling Huang, Songji Tu, Huaju Cai, Zhengyu Wu, Anbang Wei, Chengzhu Liu, Yi Hong, Xiaotong Tian, Yan Cheng, Jian Pan, Ningling Wang, Kunlong Zhang

**Affiliations:** 1 Department of Pediatrics, The Second Affiliated Hospital of Anhui Medical University, No. 678 Furong Road, Hefei City, 230601 Anhui Province China; 2 Institute of Pediatric Research, Children’s Hospital of Soochow University, No. 92 Zhongnan Street, SIP, Suzhou City, 215003 China

**Keywords:** FYB1, CAPG, AML, Proliferation, Apoptosis, Prognosis

## Abstract

Acute myeloid leukemia (AML) is a rare and heterogeneous disease. Over the past few decades, patient prognosis has improved with continuous improvements in treatment, but outcomes for some patients with primary drug resistance or relapse after treatment remain poor. Additional therapies to improve outcomes for these patients are urgently needed. FYB1 expression differs substantially between AML tissues and normal tissues. High FYB1 expression is correlated with poorer overall survival (OS), indicating that FYB1 may regulate AML progression. Therefore, understanding the effect of FYB1 on AML could improve the success rate of therapeutic approaches and prognosis for patients with AML. In this study, through analysis of large databases and both in vivo and in vitro experiments, we assessed the expression and role of FYB1 in AML and the relationship of FYB with patient prognosis. Downstream targets of the FYB1 gene were analyzed by RNA-seq. Database mining and in vitro experiments were used to further clarify the effect of the downstream target gelsolin-like actin-capping protein (CAPG) on AML cells and its relationship with patient prognosis. FYB1 expression was significantly higher in AML tissue and corresponded with a poor prognosis. FYB1 knockdown inhibited AML cell proliferation, promoted cell apoptosis, reduced cell adhesion capability and significantly reduced the tumor formation rate in mice. In addition, FYB1 knockdown induced a notable decrease in CAPG expression. The suppression of CAPG significantly inhibited cell proliferation and increased cell apoptosis. The conclusions of this study underscore the pivotal role of the FYB1/CAPG axis in promoting AML. We propose that the FYB1/CAPG axis could serve as a new thread in the development of therapeutic strategies for AML.

**Supplementary Information:**

The online version contains supplementary material available at 10.1007/s11010-024-04992-4.

## Introduction

Acute myeloid leukemia (AML) is a serious blood disorder caused by the rapid clonal expansion of myeloid blasts in the bone marrow, peripheral bloodstream, or tissues beyond the marrow [1]. The characteristics of AML include impaired maturation and differentiation of immature myeloid cells, unregulated cell proliferation, presence of abnormal blasts in peripheral blood, leading to anemia and susceptibility to infections. [2]. The predominant strategies for managing AML include chemotherapeutic treatment, precision therapy, and the transplantation of hematopoietic stem cells [3, 4]. Patient prognosis varies based on factors such as patient age and race, gene expression and epigenetic abnormalities [5, 6]. Only 24% of adults survive beyond five years after diagnosis. The prognosis of children with AML is better than that of adults with AML; children have an average 5-year survival rate that is approximately 65–70%. Additionally, recurrence, drug resistance, and drug side effects remain problematic [7]. Thus, it is crucial to investigate the complex and subtle variations in the molecular mechanisms that support the progression of AML. Through further research, we may uncover previously undiscovered treatment methods, propelling therapeutic advancement.

FYB1, also called adhesion and degranulation-promoting adapter protein (ADAP) and Fyn-binding protein (FYB), is a cytoplasmic adapter protein with a molecular weight ranging from 120 to 130 kD. [8, 9]. The FYB1 gene is located on the short arm of human chromosome 5 and has 14 transcription variants and 2 paralogous genes. FYB1 is expressed in platelets, T cells, natural killer cells, myeloid cells, and dendritic cells [10] and plays a role in cellular migration, proliferation, stimulation, and cytokine synthesis [11]. FYB1 is a binding partner of the cytoplasmic adapter protein SLP-76 [12] and the Src family kinase FYB [13], which regulates the activation, adhesion, proliferation and differentiation of T cells [14]. FYB1 is also involved in platelet activation and controls the production of interleukin-2 [15, 16]. FYB1 plays a crucial role in congenital autosomal recessive thrombocytopenia (CARST) [17], which manifest as abnormalities in platelet number and function, leading to an increased risk of bleeding. FYB1 also plays a role in autoimmune encephalomyelitis [18]. Addombati et al. reported an association between FYB gene polymorphism and SLE; specifically, 2 out of 10 single nucleotide polymorphisms in the FYB1 gene were associated with SLE susceptibility [19]. FYB1 is differentially expressed in cutaneous T-cell lymphoma (CTCL) and benign skin biopsy, with FYB significantly upregulated in advanced CTCL; therefore, the differential expression of FYB1 may help predict the risk of this disease [20]. Based on a study conducted by Wang et al., after CD8+ T cells were depleted of the signaling molecules ADAP and SKAP55, the upregulation of PD-1 expression induced by antigen stimulation blockade significantly enhanced the ability of CD8+ cytotoxic T lymphocytes (CTLs) to kill tumor cells [21]. Additionally, FYB overexpression is associated with breast cancer metastasis and breast cancer recurrence and has been identified as a potential marker for ductal carcinoma in situ (DCIS) and infiltrating ductal carcinoma (IDC) in the breast; thus, FYB exhibits potential for breast cancer diagnosis and prevention [22]. Nevertheless, the role, activity, and molecular mechanism of FYB1 in hematological tumors are still not understood. In T-ALL, FYB1 was discovered to operate as a super-enhancer (SE)-driven gene in our prior study. Further mining of the CCLE database found that the FYB1 gene is among the top two most highly expressed genes within T-ALL and AML. Our prior in vitro and in vivo experiments have provided evidence that the knockdown of FYB1 can impede the proliferation of T-ALL cells, induce apoptosis, and subsequently increase the survival rate of mice. These effects were observed to occur via modulation of the FYB1-IGLL1 axis [23]. Based on our previous research, we observed that FYB1 displays the highest expression in T-ALL, followed by AML. Therefore, recognizing FYB1's role as a pro-oncogene, we embarked on investigating its implication in AML and elucidating its potential mechanisms. To provide a comprehensive understanding of the role of FYB1 in AML, we used large databases–the CCLE database, SangerBox platform (http://sangerbox.com), and GEPIA2 tool (http://gepia2.cancer-pku.cn) to analyze the expression and correlation of FYB1 with the prognosis of AML. The findings demonstrated substantial differences in FYB1 expression between AML tissues and normal tissues. In addition, elevated FYB1 expression and decreased overall survival (OS) was significantly correlated among individuals with AML, suggesting the regulatory role of FYB1 in the growth of AML cells.

Gelsolin-like actin-capping protein (CAPG) is a component of the gelsolin family [24]. In contrast to other members of the gelsolin superfamily, the CAPG is located in both the cytoplasmic and nuclear compartments [25]. A main component of the cytoskeleton, CAPG participates in biological processes, including cell migration and morphological maintenance [26]. The CAPG gene is widely expressed in the lungs, esophagus, bone marrow, skin, stomach, and bladder. Furthermore, the CAPG gene is strongly correlated with the development and emergence of cancers. In hepatocellular carcinoma (HCC), the level of CAPG gene expression showed a strong correlation with both the incidence and prognostic outcomes of HCC. Findings have indicated that the CAPG gene facilitates the growth and dissemination of HCC cells while concurrently impeding apoptosis in these cells [27, 28]. In addition, high CAPG gene expression is associated with HCC malignancy and metastasis. According to reports, high CAPG expression is correlated with a worse prognosis in CRC patients [29]. CAPG is associated with the occurrence and prognosis of glioma [30], cell apoptosis and the proliferation of prostate cancer [31]. High CAPG expression is linked to breast cancer aggressiveness and prognosis and may encourage tumor cell growth and dissemination [32]. CAPG can additionally serve as a marker for the bone metastasis of breast tumors [33]. Additionally, CAPG can regulate the resistance and sensitivity of cancer cells to chemotherapeutic drugs [34]. Elevated CAPG expression has been demonstrated to accelerate the expansion and spread of lung cancer cells and has been linked with lung cancer malignancy and prognosis [35]. Recent research has found that the CAPG gene is related to SEs unique to AML. The interaction between CAPG and the NF-κB family transcription factor region leads to the activation of downstream genes, thereby promoting the advancement of AML. These study results indicate that the CAPG gene may promote tumor growth and have an important function in AML [36].

This study definitively establishes the high expression of FYB1 in AML patients and its correlation with overall survival (OS) rates in AML patients. Through in vivo and in vitro experiments, it has been demonstrated that downregulation of FYB1 can inhibit AML cell proliferation, induce apoptosis, and decrease adhesion ability. Furthermore, we also found a correlation between decreased FYB1 levels and reduced expression of CAPG in AML cells. All these findings collectively suggest that the FYB1-CAPG axis plays a crucial role in the progression of AML and may represent a promising therapeutic target for AML treatment.

## Materials and methodology

### Cell culture

The human AML cell lines U937, HEL, MV4-11, Kasumi-1, HL-60, THP-1, and K562 were grown in RPMI medium provided by Thermo Fisher Scientific supplemented with 10% fetal calf serum from Biological Industries and 1% penicillin‒streptomycin from Beyotime. Cells were cultured at 37 °C under a 5% CO_2_ environment. Preventive measures were constantly taken to check for mycoplasma contamination in the cell lines. Short tandem repeat (STR) analysis was conducted at regular intervals to confirm the identity of all the cell strains.

### Proliferation and viability assays

U937, MV4-11, and Kasumi-1 cells were carefully placed into 96-well plates before subsequent experiment. Following the guidelines provided by Dojindo Molecular Technologies, Tokyo, Japan, we used a Cell Counting Kit-8 (CCK-8) to evaluate cell survival rates. To ensure the accuracy and consistency of our data, each measurement was collected in triplicate, and the experiment was replicated at least three times.

### RNA preparation and real-time PCR

We employed TRIzol solution to extract total RNA, which was then transcribed into cDNA using a cDNA reverse transcription kit (4368813, Applied Biosystems). We used LightCycler®480 SYBR Green I Master Mix (cat. No. 04707516001) to conduct real-time fluorescent quantitative PCR. The sequences of the primers used in this study can be found in Supplementary Table 1.

### Lentivirus preparation and infection

In our study, we utilized the lentiviral vector pLKO.1-puro (sourced from IGE Biotechnology Ltd., China) to generate shRNAs targeting the FYB1 and CAPG genes (details in Supplementary Table 2). We also inserted the coding sequence (CDS) of FYB1 tagged with HA into the same lentiviral vector. To produce lentiviruses, we obtained envelope and packaging plasmids (pMD2.G: #12,259; psPAX2: #12,260) from Addgene. The lentiviruses were then cotransfected with the transfer plasmid into 293FT cells using polyethylenimine (linear MW 25,000 Da, 5 mg/mL, cat. No. 23966–1; Polysciences, USA). After incubation for 6 h, the medium was replaced. Virus-containing supernatants were harvested after 48 h and cleared with a 0.45-μm filter. Leukemia cells were then exposed to the lentivirus in the presence of polybrene (Sigma‒Aldrich) for 24 h. Next, we used puromycin (Sigma‒Aldrich) to establish stable AML cell lines. Additionally, we prepared the pMX-Luc-G418 plasmid and cotransfected it with pMD.2G and psPAX2 into 293FT cells to generate lentiviruses. U937 cells were transfected, and those cells that stably expressed Luc were selected with G418.

### Soft agar colony formation assay

RPMI1640 medium (2 ×) supplemented with 20% fetal calf serum, 2 × penicillin, and streptomycin was prepared and filtered using a 0.22-µm filter. In each well of a 6-well plate, a 1.5-mL mixture of 1.2% agarose gel and complete medium in a 1:1 ratio was added. The plate was then kept at room temperature to promote gelation of the mixture. After lentiviral transfection, cells were applied (3000/well). The upper gel contained equal volumes of 0.7% agarose gel and complete culture medium (with 3000 cells). Each well was filled with 1.5 mL of medium. After cultivation, the single colony formation rate was calculated.

### RNA-seq

Utilizing an established protocol from Novogene (Beijing, China), we performed RNA sequencing. A library was prepared from total RNA via cDNA synthesis, followed by next-generation sequencing. Following this, HISAT was used to map the raw reads postfiltering to obtain refined reads. We have archived the raw data from this study in the NCBI website (https://www.ncbi.nlm.nih.gov) under accession number PRJNA1019463.

### Adhesion assay

Fibronectin (Sigma, MO, USA) was coated onto a 24-well plate. MV4-11, Kasumi-1, and U937 cells (1 × 10^6^ cells per well) were seeded into the 24-well plate. Subsequently, the cells were allowed to adhere for 1 h at 37 °C. Unbound cells were gently washed away with PBS, and the bound cells were fixed with 4% paraformaldehyde solution. Then, the cells were stained with Giemsa stain (Beyotime, China) for 30 minutes, followed by gentle washing with PBS 1 to 2 times. Finally, the cells were placed under a microscope for imaging.

### Cell apoptosis

AML cells (U937, MV4-11, and Kasumi-1) infected with lentivirus were collected. Next, the proportion of cell apoptosis was determined using a cell apoptosis assay kit (556420, BD, USA) according to the manufacturer's instructions. Analysis of all cells was performed via flow cytometry on a flow cytometer (Beckman Gallios; AT49550).

### Western blot analysis

RIPA buffer was used to lyse U937, MV4-11, and Kasumi-1 cells. Following sonication, the supernatant containing the total protein was collected by centrifugation, and a BCA kit from Thermo Fisher Scientific was used to quantitatively measure the protein content. Antibodies against the following were used in this study: cleaved caspase-3 (cat. No. 9661S, Cell Signaling Technology), c-Myc (cat. No. 9402, Cell Signaling Technology), HA (cat. No. ab9110; Abcam), BCL-2 (cat. No. 15071 Cell Signaling Technology), PARP (cat. No. 9542, Cell Signaling Technology), and GAPDH (cat. No. 5174, Cell Signaling Technology). GAPDH served as an internal control. ImageJ was used to quantize the bands.

### Preparation of an in vivo xenograft leukemia model

All experimental NSG mice were procured from Shanghai Model Organisms Center, Inc. After approximately a week of care and acclimatization, the mice successfully adapted to their living conditions. They were equally assigned to two groups, namely the sh-NC group and the sh-FYB1#2 group, and respectively injected via the tail vein with 5 × 10^5^ U937-Luciferase cells, each exhibiting a cell viability greater than 90%. Throughout the experiment, we sporadically utilized the fluorescence imaging system (BERTHOLD, Germany) to gauge the tumor burden within the mice. The animal experiments were approved and licensed by the Animal Care and Use Committee of the Children's Hospital of Soochow University (CAM-SU-AP#: JP-2018-1).

### Statistical analysis

Statistical analysis was performed using GraphPad Prism 8.3.0 (GraphPad Software, Inc.). The statistical significance of differences between the two groups was assessed using a *t* test or one-way analysis of variance, and survival curves were generated using the Kaplan‒Meier method. Statistically significant *p* differences were defined based on *P < 0.05.

## Results

### AML patients with elevated FYB1 expression had a poor prognosis

**Fig. 1:**
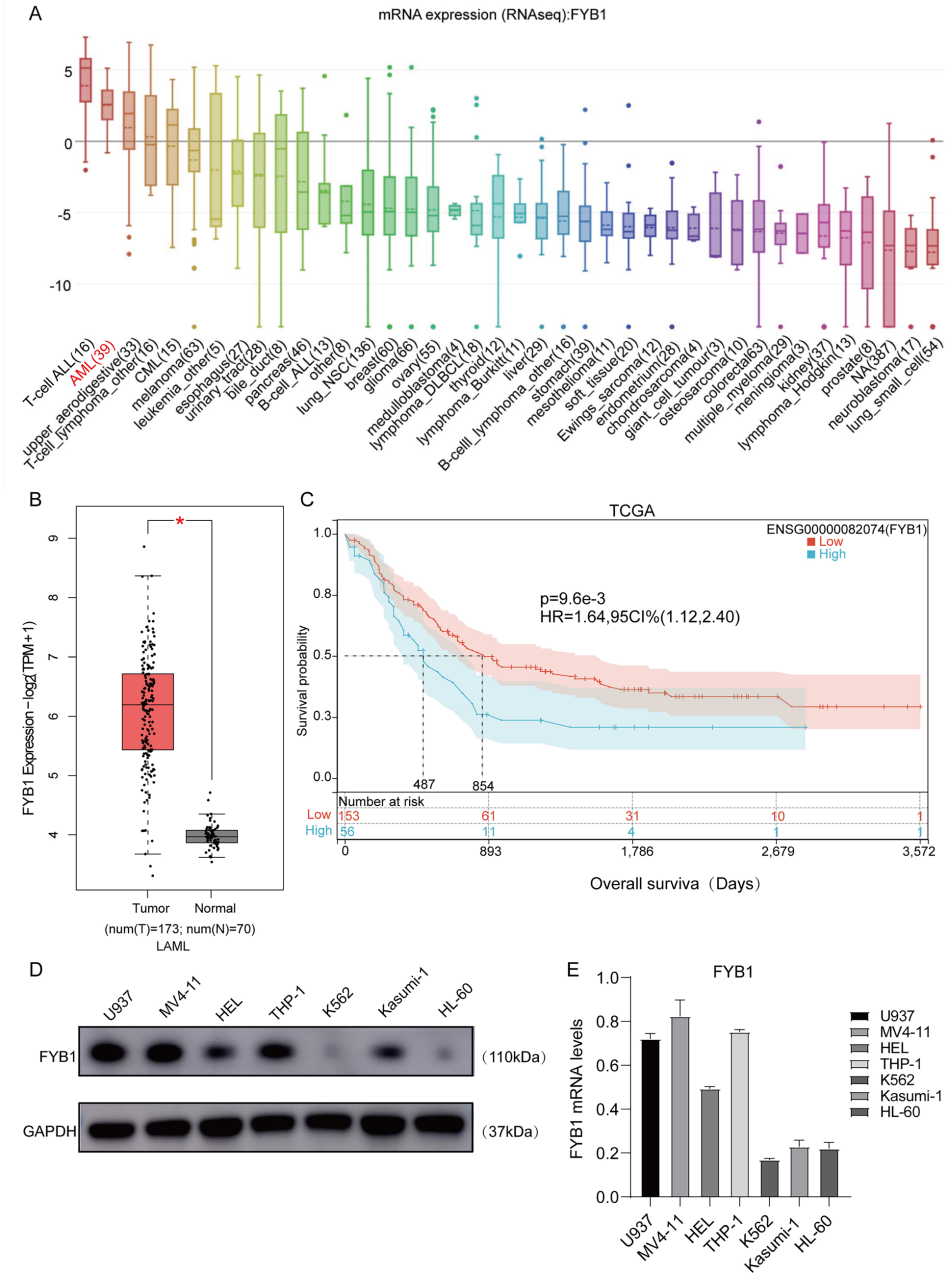
In AML, the expression of FYB1 is upregulated and correlated with patient prognosis. **A** The CCLE database shows the expression levels of FYB1 in several kinds of common cancers. **B** The expression of FYB1 in AML tissues and normal tissues was comparatively analyzed using the GEPIA2 data platform. **C** The Kaplan‒Meier method was used to detect the relationship between FYB1 expression and OS. **D**, **E** Relative expression levels of FYB1 in various cell types were analyzed by qRT‒PCR (**E**) and western blotting (**D**)

Three major tools (the CCLE database, GEPIA2 tool and SangerBox platform) were utilized to explore the role of FYB in AML. First, the CCLE database was used to examine FYB1 expression in tumor tissues. In AML, FYB1 ranked second in terms of gene expression (Fig. 1A). Mining of the GEPIA2 database revealed that FYB1 gene expression was higher in 173 AML patients than in 70 healthy subjects (Fig. 1B). In addition, Kaplan‒Meier survival analysis showed that FYB1 mRNA levels were associated with poor prognosis (Fig. 1C). Furthermore, western blotting was performed to assess the protein expression level of FYB1 in AML cell lines, and the findings revealed that FYB1 expression differed between AML cell lines. In U937, MV4-11, HEL, THP1, and Kasumi-1 cells, FYB1 expression was relatively high (Fig. 1D). Similar results were obtained by qRT‒PCR (Fig. 1E). In the GSE149237 dataset, our analysis revealed an upregulation trend in FYB1 expression in AML HSPC compared to Healthy HSPC (see Supplementary Fig. 2A). Additionally, we compared the differences in FYB1 expression between normal and cancer cell lines and found that both mRNA and protein levels of FYB1 in cancer cell lines were higher than in CD34+ HSPC (Supplementary Fig. 2C–E). In summary, the FYB1 gene exhibits significantly increased expression in AML tissues, suggesting a key role in AML cell survival.

### FYB1 promotes proliferation and affects adhesion in AML cells

**Fig. 2:**
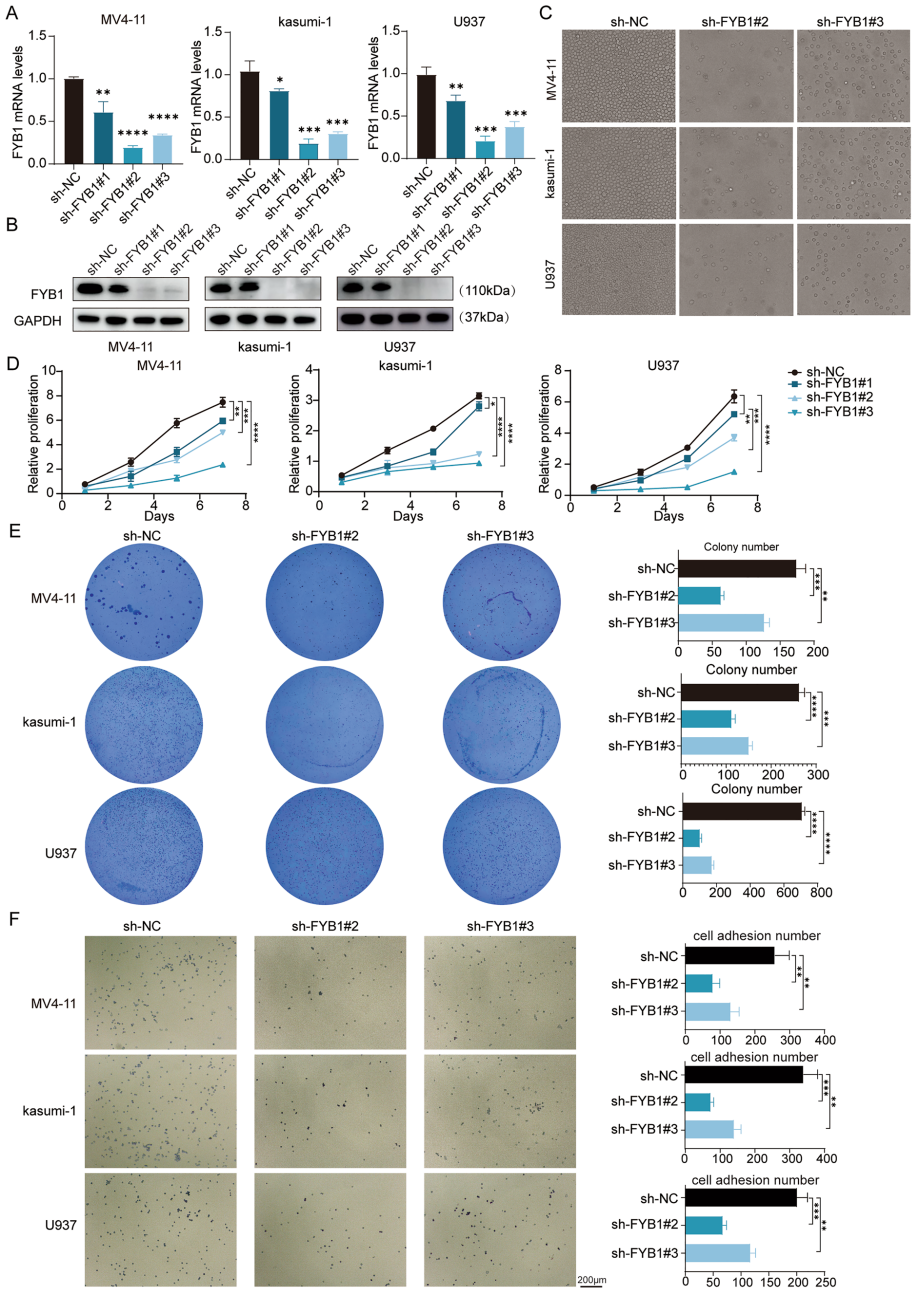
FYB1 knockdown reduced the growth, colony formation capacity, and adhesion ability of AML cells. **A**, **B** The efficiency of shRNA-mediated knockout of the FYB1 gene in MV4-11, Kasumi-1 and U937 cells was evaluated by qRT‒PCR (**A**) and western blotting (**B**). **C** Knockdown of FYB1 dramatically decreased the proliferation of MV4-11, Kasumi-1 and U937 cells, as assessed by imaging. **D** The proliferation of AML FYB1-knockdown cells was analyzed using the CCK-8 assay. **E** Colony formation was assessed by analysis of the number of colonies consisting of FYB1-knockdown AML cells. **F** Knockdown of FYB1 significantly reduced the adhesion ability of MV4-11, Kasumi-1, and U937 cells

To study the specific role of FYB1 in AML cells, three shRNAs targeting the FYB1 gene were used to silence FYB1 in AML cell lines. qRT‒PCR and western blotting (Fig. 2A, B) were used to analyze FYB1 gene and protein levels after knockdown in the AML cell lines. Microscopy revealed significantly fewer cells in the sh-FYB1#2 and sh-FYB1#3 groups than in the sh-NC group (Fig. 2C). The cell survival curves showed that FYB1 knockdown decreased AML cell viability over time (Fig. 2D). In addition, the colony formation capacity and adhesion ability of MV4-11, Kasumi-1, and U937 cells with FYB1 knockdown were reduced (Fig. 2E, F).

### FYB1 inhibits the apoptosis of AML cells

**Fig. 3:**
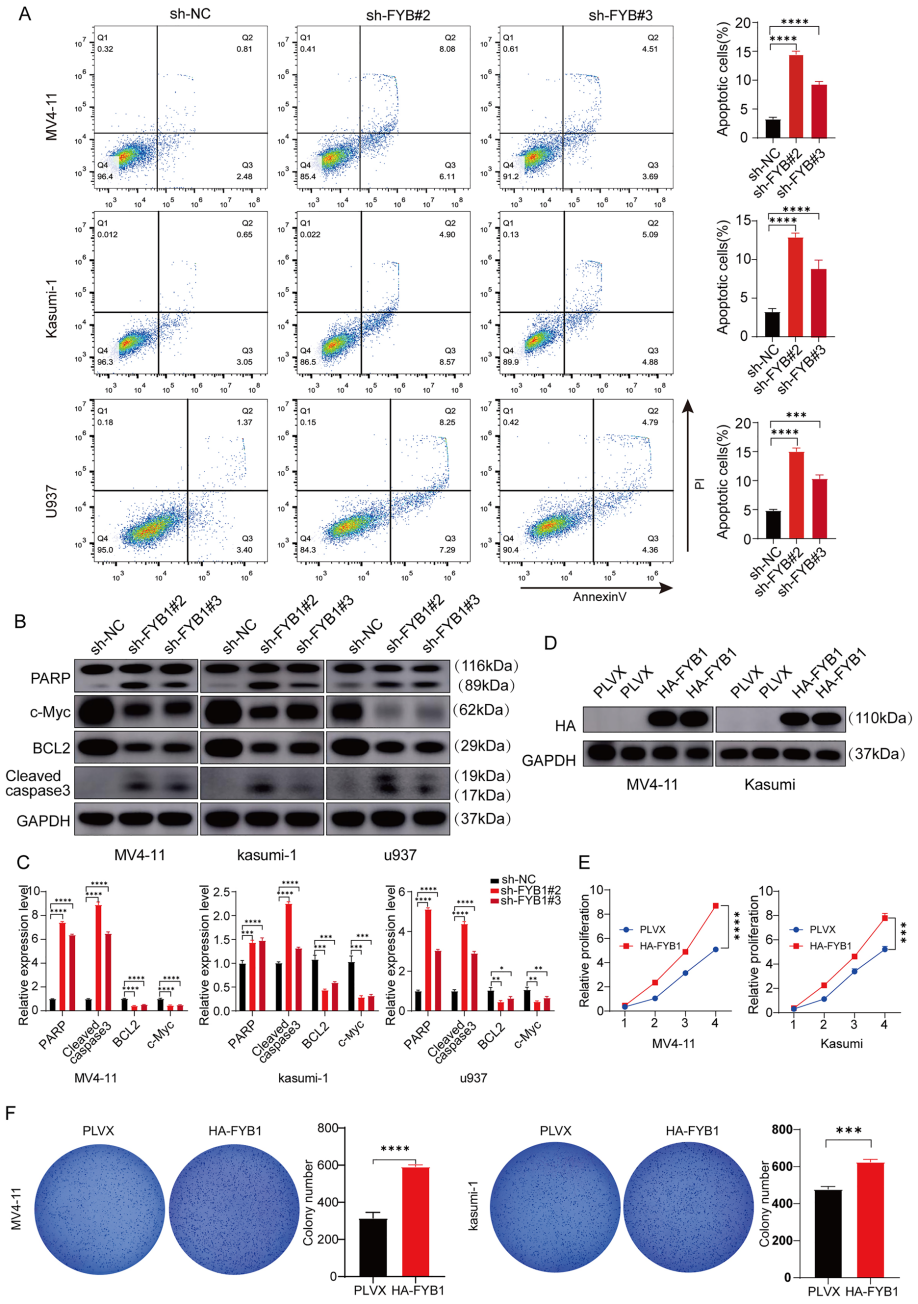
FYB1 knockdown promoted cell apoptosis in vitro, and FYB1 upregulation promoted the growth of AML cells. **A** Flow cytometry analysis of AML FYB1-knockdown cells. **B** Western blot analysis of PARP, c-Myc, Bcl-2, and cleaved caspase-3 (c-caspase-3) expression in FYB1-knockdown AML cells. **C** Gray value analysis of the protein bands **B** was performed using ImageJ. **D**–**F** Upregulation of FYB1 promoted the growth of MV4-11 and Kasumi-1 cells and their colony formation potential

Next, the impact of FYB1 on AML cell apoptosis was examined. Flow cytometry demonstrated that FYB1 gene knockdown increased AML cell death relative to that in the control group (Fig. 3A). In contrast to those in the sh-NC group, the expression levels of cleaved PARP and cleaved caspase-3 were elevated in AML cells in the sh-FYB1#2 and sh-FYB1#3 groups (Fig. 3B, C). Additionally, the antiapoptotic proteins Bcl-2 and c-Myc were downregulated in FYB1-knockdown cells (Fig. 3B, C). To further elucidate the function of FYB1 in AML cells, an FYB1-specific overexpression plasmid was transfected into cells and validated by western blotting (Fig. 3D). Cell survival curves and CCK-8 data showed that FYB1-overexpressing AML cells proliferated more than control cells (Fig. 3E). MV4-11 and Kasumi-1 cells expressing high levels of FYB1 created more colonies than control cells (Fig. 3F). Taken together, these findings indicate that FYB1 knockdown induced AML cell apoptosis and that the overexpression of FYB1 promoted AML cell growth.

### FYB1 knockdown significantly inhibited the growth of AML cells in vivo

**Fig. 4:**
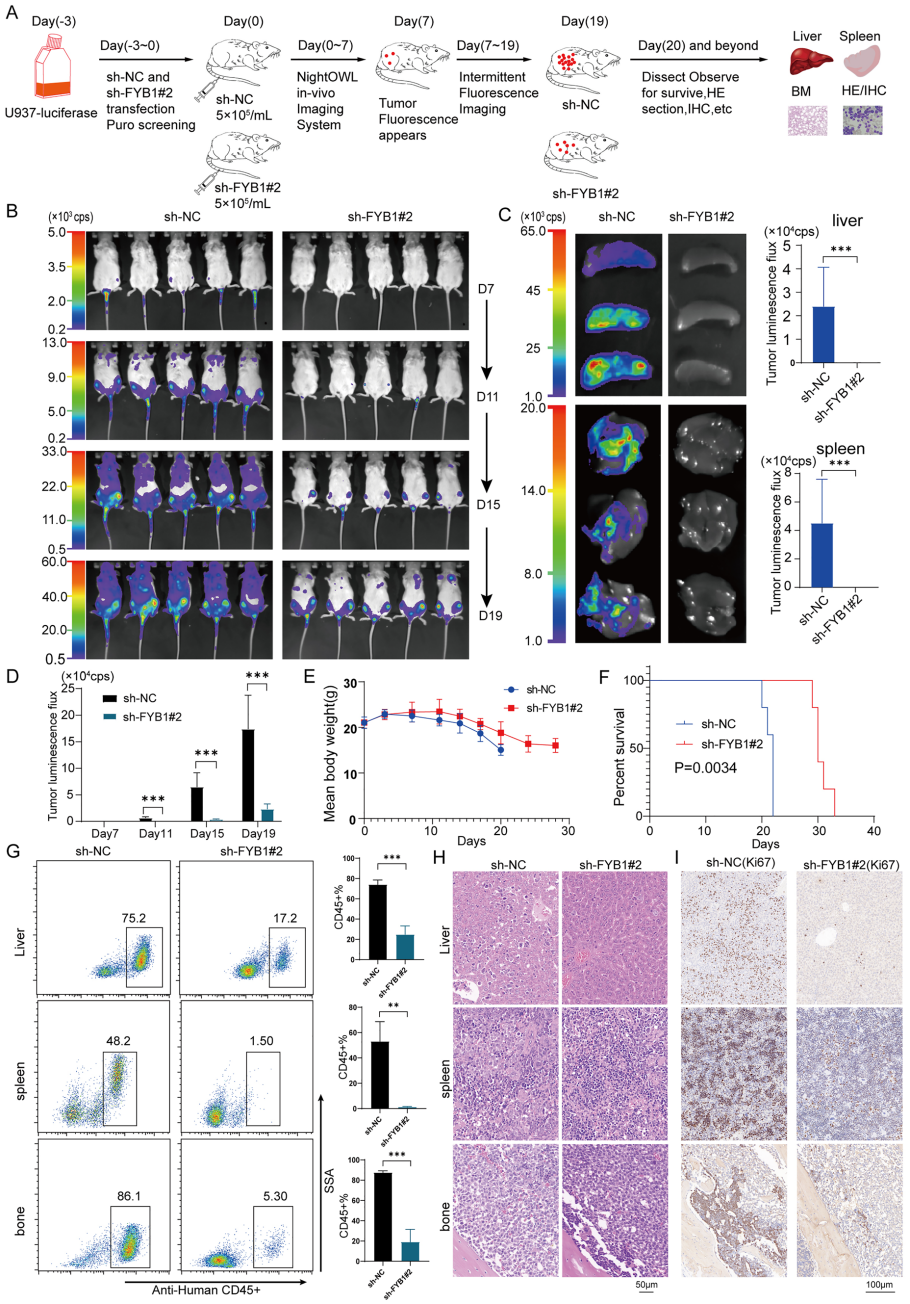
Knockdown of the FYB1 gene significantly attenuated AML tumorigenesis in NSG mice. **A** Flowchart of the animal experiment. **B** We used the NightOWL imaging system to observe the development of leukemia in mice. The fluorescence values are shown in a bar graph (**D**). **C** Fluorescence imaging of the liver and spleen in the two groups of mice; fluorescence values are shown in bar graphs. **E** Changes in body weight in the control group and FYB1-knockout group are shown. **F** Mice in the FYB1-knockdown group exhibited substantially greater survival (P = 0.0034). **G** The CD45 ratio in the liver, spleen, and bone marrow was detected using flow cytometry; the results are shown in histograms. **H** and **I** Typical H&E staining and immunohistochemistry images of the mouse liver, spleen, and bone marrow

In vitro, the knockdown of FYB1 inhibited AML growth and promoted AML apoptosis. Therefore, the effect of FYB1 was studied in an immunodeficient mouse model. U937-luciferase cells with or without FYB1 knockdown were injected into NSG mice via the tail vein (Fig. 4A). The leukemia burden was measured via in vivo fluorescence imaging. As shown in Fig. 4B and D, compared with that of cells in the control group, FYB1 knockdown drastically reduced the proliferation of U937-luciferase cells in vivo at four random time intervals. Accordingly, liver and spleen fluorescence signals were considerably lower in mice injected with FYB1 U937-luciferase cells than mice in the control group (Fig. 4C). The liver, spleen, and bone marrow had considerably fewer FYB1-knockdown U937-luciferase cells than did those of the control group (Fig. 4G). In addition, H&E staining and immunohistochemistry (Ki67) demonstrated that the number of FYB1-knockdown U937-luciferase cells in the liver, spleen and bone marrow was considerably reduced (Fig. 4H, I). Furthermore, the survival rate of FYB1-knockdown mice was higher (30.6 ± 1.5166 days) (P = 0.0034) than that of mice in the control group (21.4 ± 0.8944 days) (Fig. 4F). No significant difference in body weight was detected (Fig. 4E). These results indicate that FYB1 knockdown dramatically slowed AML cell proliferation in vivo.

### FYB1 activates CAPG in AML cell lines

**Fig. 5:**
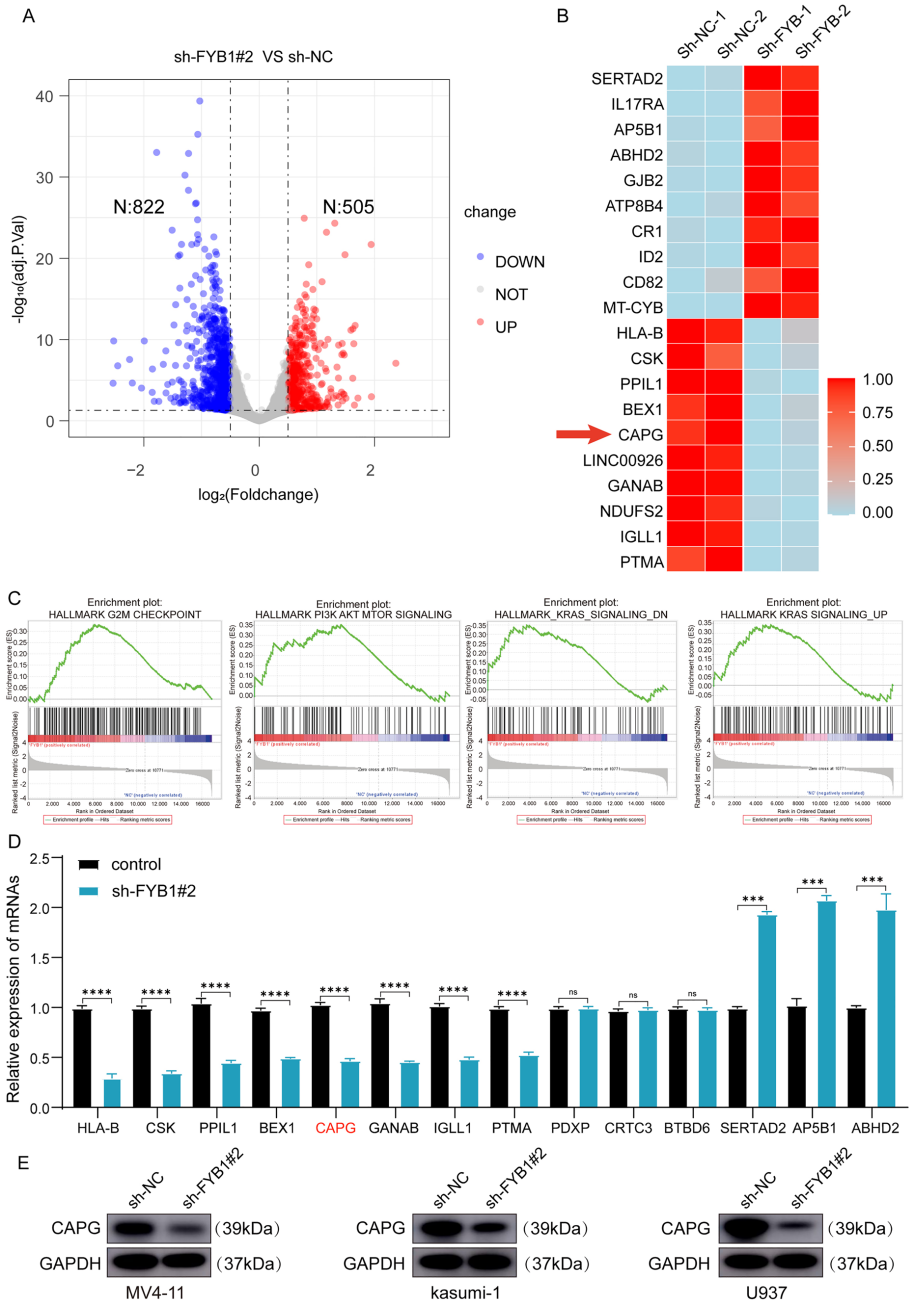
FYB1 activated CAPG in AML cells. **A** Volcano map showing the levels of differentially expressed genes in U937 cells with or without FYB1 knockdown. **B** Upregulated and downregulated genes in U937 cells after FYB1 gene knockout are shown. **C** GSEA showed that gene signatures in the G2M, PI3K AKT MTOR and KRAS pathways were enriched after FYB1 knockdown in U937 cells. **D** and **E** Changes in the mRNA expression of potential gene targets of FYB1 were verified by qRT‒PCR. The protein level of CAPG, a possible downstream target of FYB1, in FYB1-knockdown AML cells (MV4-11, Kasumi-1, and U937 cells) was analyzed by western blotting

To identify the targets through which FYB1 promotes AML cell growth, RNA-seq analysis (PRJNA1019463) was performed using U937 cells with or without FYB1 knockdown. After knockdown of the FYB1 gene, 1327 differentially expressed genes (DEGs), including 822 downregulated genes and 505 upregulated genes (P < 0.05 and Log2 (fo1d-change) > 0.5 or Log2 (fold-change) < − 0.5; Fig. 5A and Supplementary Table 3) were identified. The GSEA data also indicated that after FYB1 knockdown in U937 cells, genes in the HALLMARK G2M CHECKPOINT, HALLMARK PI3K AKT MTOR SIGNALING, HALLMARK_KRAS_SIGNALING_DN and HALLMARK KRAS SIGNALING_UP signaling pathways were enriched (Fig. 5C).. The top downregulated genes in the FYB1 group are shown in a heatmap (Fig. 5B). The RNA-seq results were validated using qRT‒PCR (Fig. 5D). Among the downregulated genes, expression of the CAPG gene was notably silenced. In addition, as confirmed through western blotting, after FYB1 knockdown, CAPG was also downregulated (Fig. 5E). Furthermore, analysis of the GSE183385 dataset (RNA-seq of splenic macrophages from WT and Adap^−/−^ mice) revealed downregulation of CAPG as well (Supplementary Fig. 1). All of these results indicate that CAPG may serve as a downstream target regulated by FYB1.

### CAPG knockdown interfered with proliferation and induced AML cell apoptosis

**Fig. 6:**
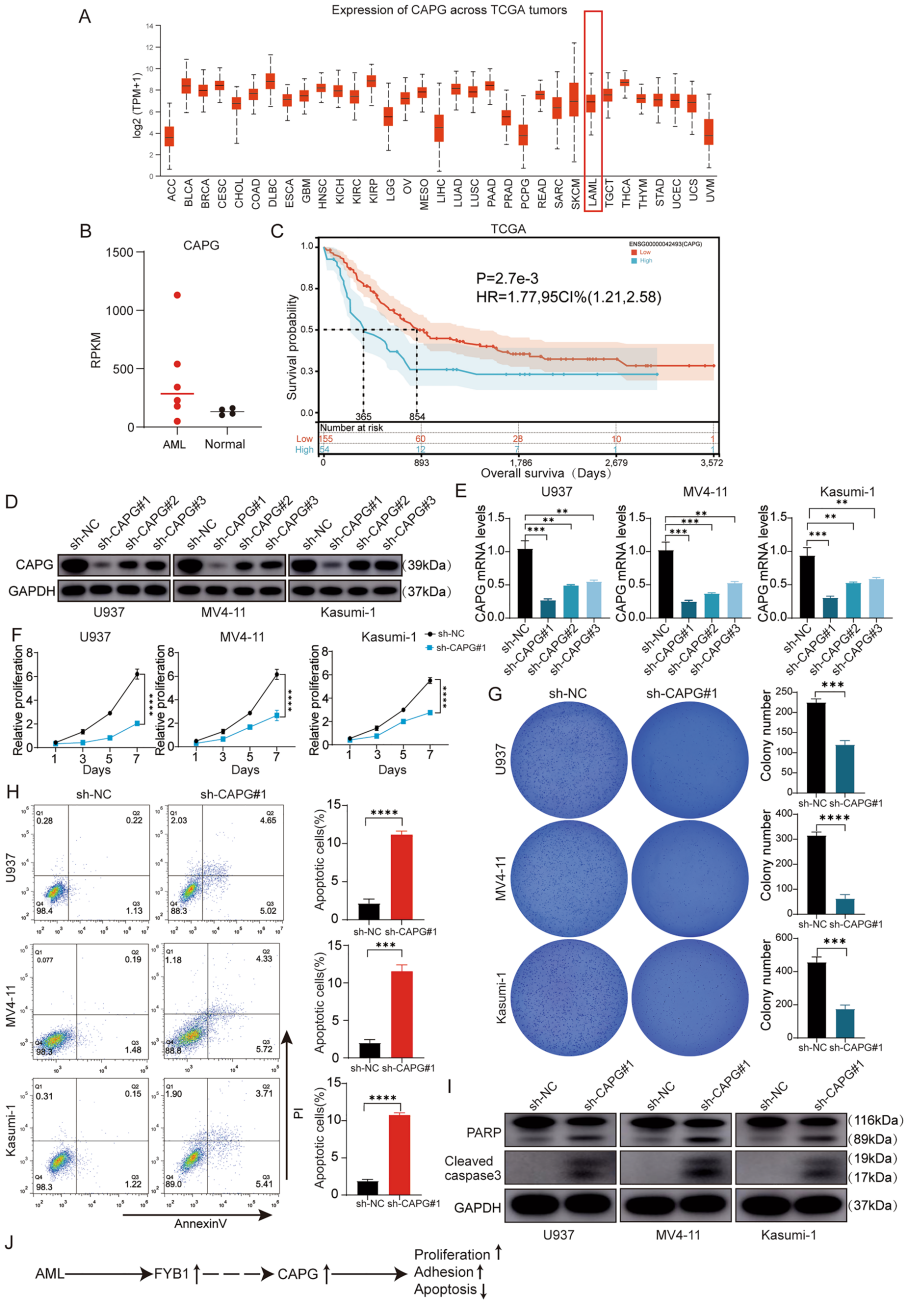
Knockdown of CAPG in AML interfered with proliferation and promoted AML cell apoptosis. **A** The expression levels of CAPG in common tumors included in the UALCAN database are shown. **B** Analysis of the RNA-seq dataset (GSE128910) showed that CAPG was highly expressed in AML patients. **C** The effect of CAPG expression on survival is shown. Kaplan‒Meier curves for CAPG were prepared using data obtained from the SangerBox database. **D** and **E** The efficiency of shRNA-mediated knockout of the CAPG gene in MV4-11, Kasumi-1 and U937 cells was evaluated by qRT‒PCR (**E**) and western blotting (**D**). **F** A CCK-8 assay was performed to assess the proliferation of CAPG-knockout AML cells using the CCK8. **G** Colony formation capacity was assessed via analysis of the number of colonies consisting of AML cells with CAPG knockdown. **H** Flow cytometry analysis of CAPG-knockdown AML cells was performed. **I** Western blot analysis for PARP and cleaved caspase-3 (c-caspase-3) in CAPG-knockdown AML cells was performed. **J** Schematic diagram of FYB1-CAPG axis regulation

To further investigate the role of CAPG in AML, the UALCAN (https://ualcan.path.uab.edu/) database was analyzed, which revealed that CAPG was moderately expressed in AML (Fig. 6A). Analysis of the RNA-seq dataset (GSE128910) showed substantially increased CAPG expression in AML patients (Fig. 6B) (healthy subjects (n = 4) and AML patients (n = 7)). In the GSE149237 dataset, our analysis revealed an upregulation trend in CAPG expression in AML HSPC compared to Healthy HSPC (see Supplementary Fig. 2B). Additionally, we compared the differences in CAPG expression between normal and cancer cell lines and found that both mRNA and protein levels of CAPG in cancer cell lines were higher than in CD34+ HSPC (Supplementary Fig. 2D–F). In addition, Kaplan‒Meier survival plots prepared by mining the SangerBox database showed that the CAPG mRNA level was correlated with a poor prognosis in AML patients (Fig. 6C). To assess the biological function of CAPG, three different shRNAs (Supplementary Table 2) were employed to knock down CAPG in three AML cell lines (U937, MV4-11, and Kasumi-1 cells). Figure 6E and D shows that qRT‒PCR and western blot verified CAPG knockdown. As shown in Fig. 6D and E, sh-CAPG#1 was the most effective in knocking down CAPG. The survival of AML cells with CAPG knockdown decreased in a time-dependent manner according to the experimental results (Fig. 6F). In addition, the colony formation capacity of sh-CAPG#1 U937, MV4-11, and Kasumi-1 cells was decreased (Fig. 6G). We then studied the effect of CAPG on AML cell apoptosis. Flow cytometry showed that CAPG gene knockdown increased the apoptosis of AML cells compared with that in the control cells (Fig. 6H). This result was also confirmed by detection of the apoptosis markers cleaved PARP and cleaved caspase-3 in AML cells in the sh-CAPG#1 group (Fig. 6I). Collectively, these results suggest that CAPG facilitates AML proliferation.

## Discussion

AML is a malignant disease characterized by aberrant proliferation of primitive and immature myeloid cells in both the bone marrow and peripheral blood. Despite considerable advancements in understanding its pathogenesis and preclinical investigations, the 5-year survival rate for AML patients remains suboptimal, at less than 40% [37]. Therefore, there is an imminent demand within clinical settings to conduct research on anti-AML medications targeting novel pathways and mechanisms. Our prior investigation revealed FYB1 as a gene distinctly governed by super-enhancers in T-ALL, exhibiting notably elevated expression levels in this condition. Through interference with FYB1, we observed a robust regulatory impact on the proliferation of T-ALL cell lines [23]. Drawing from prior CCLE studies, we noted that FYB1 expression is most pronounced in T-ALL, closely followed by AML. Consequently, considering FYB1’s role as a pro-oncogene, we embarked on investigating its involvement in AML and elucidating its underlying mechanisms.

FYB1, also recognized as the adhesion and degranulation promoting adapter protein (ADAP), remains relatively underexplored despite its pivotal role. In addition to our investigation into its implication in T-ALL as mentioned earlier, FYB1 is extensively documented for its significance in T cells. It plays a crucial role in T cell activation and functions as a linking protein in the FYN and LCP2 signaling pathways within T cells [38]. Furthermore, FYB1 expression on primary natural killer cells and interleukin-2-stimulated lymphocytes can activate these killer cells, thereby bolstering the antitumor response [10]. Moreover, the post-translational modification of FYB1 may lead to an increase in tyrosine phosphorylation by affecting the attachment of the T cell receptor [39]. Our investigation indicates that FYB1 exhibits elevated expression levels in AML, correlating with unfavorable prognoses, suggesting its potential oncogenic role. In the context of AML, silencing FYB1 demonstrates inhibitory effects on proliferation, induces apoptosis, and reduces adhesive capabilities. Conversely, FYB1 overexpression enhances proliferation. Additionally, in vivo experiments validate that silencing FYB1 prolongs mouse survival, suppresses AML cell proliferation, and infiltration. In summary, FYB1 may promote AML progression by fostering proliferation, impeding apoptosis, and enhancing adhesion.

Our RNA sequencing results uncovered that interference with FYB1 in AML cells resulted in altered expression of genes enriched in the PI3K/AKT/mTOR signaling pathway. From our literature review, we identified CAPG (Capping actin protein of gilgamesh) as a significant regulatory protein in this pathway [40], supported by experimental evidence confirming that silencing FYB1 affects CAPG expression levels. Interestingly, RNA sequencing of splenic macrophages from both WT and Adap^−/−^ mice also revealed enrichment of genes with altered expression in the PI3K/AKT/mTOR signaling pathway [41]. Furthermore, analysis of the GSE183385 dataset demonstrated downregulation of CAPG as well. We speculate that in AML, CAPG acts as a downstream regulator of FYB1 in modulating the PI3K/AKT/mTOR signaling pathway. However, the specific regulatory mechanisms are currently under further investigation. CAPG is a protein that binds to actin and is involved in processes such as cell movement, morphological changes, and signal transduction [24]. In tumor cells, the expression and function of CAPG may change. Some studies have found that CAPG is overexpressed in some tumors and is related to the invasion, metastasis, and prognosis of the tumor [26]. For example, in tumors such as breast cancer and lung cancer, high expression of CAPG may be related to the malignancy and poor prognosis of the tumor [33–35]. On the other hand, CAPG may also be involved in the movement and migration of tumor cells. The migration of tumor cells is an important step in tumor metastasis, and CAPG may affect the migration ability of tumor cells by regulating the dynamic changes of the cytoskeleton [42]. In addition, the expression and function of CAPG in different types of tumors and tumor cells may vary. The latest research results also show that CAPG can promote the development of AML by regulating NF-κB signaling pathway. This indicates that CAPG may be a viable therapeutic target for AML [36]. In our study, by interfering with CAPG, its role in AML has been confirmed, that is, low expression of CAPG will promote the apoptosis of AML cells and inhibit cell proliferation.

## Conclusion

In summary, our study verified the role of FYB1 in AML through in vivo and in vitro experiments, and confirmed that low expression of FYB1 would promote the apoptosis of AML cells. In order to explore the specific mechanism of its regulation on the apoptosis of AML cells, we conducted RNA-seq analysis, and found that the PI3K/AKT/mTOR signaling pathway had changed. In order to study the mechanism by which FYB1 regulates this signaling pathway, through literature research and examination of the differential gene expression table, we found that CAPG was significantly reduced. It is reported in the literature that CAPG is a core regulatory protein in the mTOR signaling pathway, and its expression is related to AML. Therefore, we selected CAPG as the downstream target. By interfering with CAPG, we confirmed that it can affect the survival of AML cells. In summary, this study found that the FYB1-CAPG axis may regulates the PI3K/AKT/mTOR signaling pathway, thereby affecting the survival of AML cells, and FYB1-CAPG axis is a new target we have discovered.

## Supplementary Information

Below is the link to the electronic supplementary material.

Supplementary file1 (DOCX 202 KB)

Supplementary file2 (CSV 238 KB)

## Data Availability

The study data can be found online at https://www.ncbi.nlm.nih.gov under accession number PRJNA1019463.
