# Supplementary material for: FYB1-targeted modulation of CAPG promotes AML progression
Source: Mol Cell Biochem. 2024 May 3;480(2):985–99. doi: 10.1007/s11010-024-04992-4 (PMC11836086; doi:10.1007/s11010-024-04992-4)

Supplementary Table 1：PCR primer sequence number

| HLA-B | Forward Primer | CAGTTCGTGAGGTTCGACAG | Reverse Primer | CAGCCGTACATGCTCTGGA |
| --- | --- | --- | --- | --- |
| CSK | Forward Primer | CTGTACGCGCCTCATTAAACC | Reverse Primer | CAGCATCACGTCTCCGAACTC |
| PPIL1 | Forward Primer | TGAGTTGGCTCGTCGAGGTTA | Reverse Primer | GCCATAGATAGATGCACCACC |
| BEX1 | Forward Primer | CAGCATGGAAAATGCCAACCA | Reverse Primer | ATGATGGTCATGGTGAGGGG |
| CAPG | Forward Primer | CAACATCCTGGAACGCAACA | Reverse Primer | AGCAGTTCAAGGGCAAATGG |
| GANAB | Forward Primer | TGGGGATTACCCTTGCTGTG | Reverse Primer | CCGTATGCTTCTCTGTCGCT |
| IGLL1 | Forward Primer | CGTGGTAACCCATGGCCTG | Reverse Primer | ATGAACATTCTGCAGGGGCC |
| PTMA | Forward Primer | GGAGGCTGACAATGAGGTAGA | Reverse Primer | TGGTATCGACATCGTCATCCT |
| PDXP | Forward Primer | CTGGAGACCGACATCCTCTTT | Reverse Primer | TTCTAGGCGGGAGACTCCTG |
| CRTC3 | Forward Primer | AGTTTCAGCCGTCATTTCACC | Reverse Primer | AGCACTACCATCAAATTGTCGC |
| BTBD6 | Forward Primer | GCCATGTTCTACGGAGACCTG | Reverse Primer | TCTTAGCAGCGTACAGAGTGG |
| SERTAD2 | Forward Primer | TGTCCCACATCTACCTCCACA | Reverse Primer | CTTGAGGACCGTCGAGTTTCT |
| AP5B1 | Forward Primer | TGCCTGCGAGAGCTAGAGAG | Reverse Primer | TGGGGAGACCTTATCCGTGAG |
| ABHD2 | Forward Primer | CATGCTGGAGACTCCCGAAC | Reverse Primer | CAAACACCGGACGATCACGTA |
| FYB1 | Forward Primer | GGATGTCTCAGTCAATAGCCG | Reverse Primer | GGTTCCTTGTCAGGCTTTTCC |
| GAPDH | Forward Primer | TGCACCACCAACTGCTTAG | Reverse Primer | GATGCAGGGATGATGTTC |

Supplementary Table 2：shRNA Sequence

| FYB1-sh1 | CCGGCCAAATGTTGACCTGACGAAACTCGAGTTTCGTCAGGTCAACATTTGGTTTTTTGAATT |
| --- | --- |
| FYB1-sh2 | CCGGGCTTCAAGCAAGGAGAGCAAACTCGAGTTTGCTCTCCTTGCTTGAAGCTTTTTTGAATT |
| FYB1-sh3 | CCGGGCCATCTCTTCACAGTGTAAACTCGAGTTTACACTGTGAAGAGATGGCTTTTTTGAATT |
| CAPG-sh1 | CCGGGCATTTCACAAGACCTCCACACTCGAGTGTGGAGGTCTTGTGAAATGCTTTTTGAATT |
| CAPG-sh2 | CCGGGCTGATATCTGATGACTGCTTCTCGAGAAGCAGTCATCAGATATCAGCTTTTTGAATT |
| CAPG-sh3 | CCGGGAGTCCCATCTTCAAGCAATTCTCGAGAATTGCTTGAAGATGGGACTCTTTTTGAATT |

**Supplementary Figure 1**

Differential expression plot of CAPG

Analysis of the RNA-seq dataset (GSE183385) revealed significant downregulation of CAPG after knocking down ADAP.


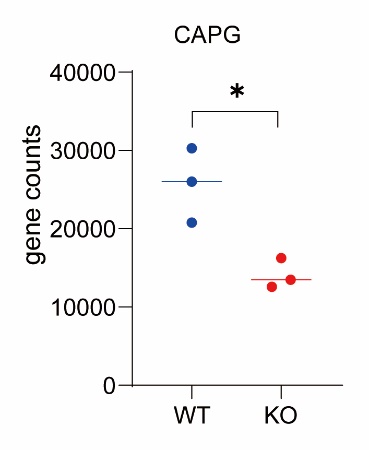


Supplementary Figure 2

FYB1 and CAPG gene expression between normal and cancer cell lines.

(A-B) The expression levels of FYB1 and CAPG in GSE149237.

(C-D) The protein expression levels of FYB1 and CAPG between CD34+ HSPC and cancer cell lines.

(E-F) The mRNA levels of FYB1 and CAPG between CD34+ HSPC and cancer cell lines.


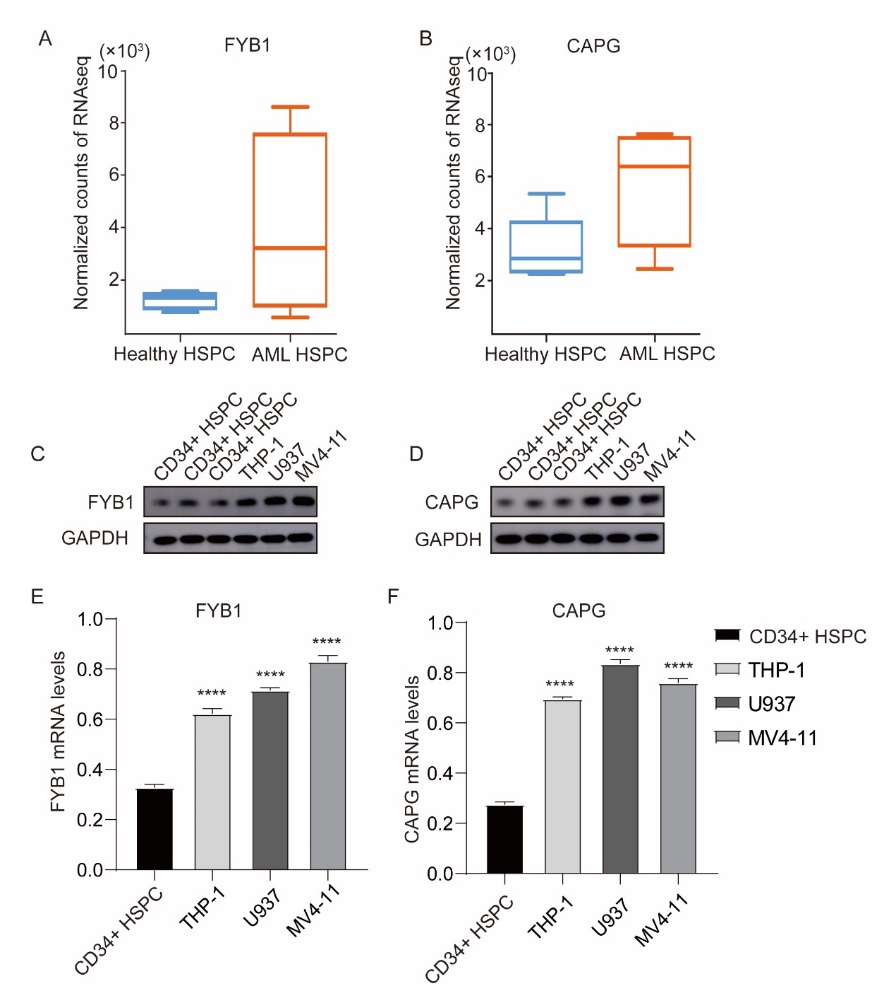

Supplement: Supplementary file 1 — Supplementary file1 (DOCX 202 KB) [file 11010_2024_4992_MOESM1_ESM.docx]
